# Diagnostic utility of apparent diffusion coefficient values in cervical cancer staging: A retrospective study

**DOI:** 10.1016/j.ejro.2026.100744

**Published:** 2026-04-03

**Authors:** Peyman Kamali Hakim, Fahimeh Zeinalkhani, Soroush Alaeddini, Saeed Mohammadzadeh, Fatemeh Mahdavi Sabet, Hadise Zeinalkhani, Mahdi Hazratgholi

**Affiliations:** a Advanced Diagnostic and Interventional Radiology Research Center (ADIR), Tehran University of Medical Sciences, Imam Khomeini Hospital, Tehran, Iran; b Department of Radiology, Tehran University of Medical Sciences, Imam Khomeini Hospital, Tehran, Iran; c Faculty of Medicine, Tehran Medical Sciences Branch, Islamic Azad University, Tehran, Iran; d Department of Radiology, Shahid Beheshti University of Medical Sciences, Tehran, Iran

**Keywords:** Cervical cancer, Diffusion-Weighted Imaging, Apparent Diffusion Coefficient

## Abstract

**Background and purpose:**

Accurate staging is critical for therapeutic planning, which directly determines prognosis, as management shifts from surgical resection in early stages to concurrent chemoradiation in advanced disease. We aimed to evaluate diagnostic utility of apparent diffusion coefficient (ADC) in staging of cervical cancer.

**Material and methods:**

A retrospective study of 146 consecutive patients with histopathologically confirmed cervical cancer, who underwent pelvic MRI with DWI at our tertiary care center was conducted. Two independent radiologists performed tumor staging according to FIGO staging classification and regions of interest were drawn on ADC maps. Spearman’s rank correlation was employed to evaluate the relationship between ADC values and age, stage, and tumor size. Diagnostic performance parameters, including sensitivity, specificity, and area under the curve (AUC) were assessed.

**Results:**

Median ADC values (×10⁻⁶ mm²/s) decreased significantly with disease progression: stage I, 966 (IQR: 182); stage II, 830 (IQR: 287); stage III, 811 (IQR: 230); and stage IV, 750 (IQR: 231); p < 0.05. Moreover, median ADC values differed significantly between patients with and without parametrial, vaginal, and lymph node involvement. (p-values <0.05). ADC values distinguished tumors of ≥ 4 cm from smaller ones with a sensitivity and specificity of 82.1% and 47.8%, respectively. Parametrial and lymph node involvement were detected using ADC values with sensitivities of 85.0% and 68.8% and specificities of 58.0% and 57.1%, respectively.

**Conclusion:**

ADC values could be a reliable tool for facilitating more accurate cervical cancer staging and could inform clinical management and decision-making on therapy intensification or de-escalation.

## 1 Introduction

According to the world health organization (WHO), cervical cancer is the fourth most prevalent cancer among the female population. The mortality rate of cervical cancer is higher in developing countries as 94% of 350,000 cancer-related deaths happened in low- and middle-income countries. The higher rate of cervical cancer mortality is contributed to by insufficient coverage of human papilloma virus (HPV) vaccination and cervical cancer screening programs [1]. Cervical cancer mortality is strongly associated with limited access to healthcare and delayed treatment, thus early detection could improve prognosis and patient survival [2].

Selection of proper therapeutic intervention can decrease mortality and improve prognosis, with appropriate treatment being strongly tied to the stage of cancer [3]. One of the most acquired staging tools is the International Federation of Gynecology and Obstetrics (FIGO) staging classification for cervical cancer. According to the latest version of the FIGO classification, there is a great emphasis on the importance of imaging modalities such as MRI in the evaluation of tumor size and lymph node involvement which ensures optimization of cancer staging and appropriate treatment [4].

Among MRI techniques, diffusion-weighted imaging (DWI) shows the variance of water molecules' mobility, tissue cellularity and membrane integrity [5]. The rate of this parameter is measured by the apparent diffusion coefficient (ADC). Additionally, DWI has revealed promising results in differentiating malignant tumors from benign ones as they typically have a dense cellular structure which can result in lower water molecule motion and consequently lower ADC values [6]. Furthermore, by acquiring ADC mapping, the boundary between normal and malignant tissues can be differentiated with high sensitivity and specificity in cervical cancer patients [7]. Multiple studies have emphasized the role of ADC values as a biomarker for diagnosis and staging as well as prognosis and recurrence of tumors in various areas such as the brain, head, neck and pelvis [8], [9]. Specifically in gynecology, there is an established role for DWI for differentiating high-grade endometrial cancers from low-grade ones. In addition, DWI is more accurate than T2-weighted images for tumor size assessment. It is also useful for the prediction of treatment success after chemotherapy in patients who are not fit for surgery [10].

Non-contrast MRI techniques, including DWI, offer an advantage by eliminating the risk of adverse effects associated with contrast agents. Considering the increasing prevalence of chronic kidney disease, gadolinium-based contrast agent exposure can increase the risk of gadolinium toxicity and its complications such as nephrogenic systemic fibrosis in certain conditions such as pregnancy and renal disfunction [11]. Therefore, non-contrast sequences like DWI can be used as complementary tools that expand diagnostic options and may help reduce, but not replace, contrast use in select clinical populations.

The capability of DWI and ADC sequences to differentiate tissue based on cellular density presents an opportunity to enhance gynecological oncologic imaging. A study showed that low primary tumor ADC and a high myometrium-to-tumor ADC ratio are powerful, stage-independent predictors of reduced disease-specific survival, highlighting their potential as quantitative biomarkers for identifying high-risk phenotypes in cervical cancer [12]. Another retrospective study of 57 cervical cancer patients undergoing MRI, Rizescu et al. found that tumoral ADC values (mean 0.79 × 10⁻³ mm²/s) were significantly lower than in normal cervical tissue (mean 1.59 × 10⁻³ mm²/s) and decreased with higher FIGO stage, larger tumor size, and pelvic lymph node involvement. These findings underscore ADC's potential as a quantitative biomarker reflecting tumor aggressiveness [13].

While prior studies have reported associations between ADC values and FIGO stage in cervical cancer, a clear consensus on ADC's role in preoperative staging remains lacking, and no prior work has thoroughly evaluated ADC performance across a comprehensive set of prognostically relevant tumor characteristics including, tumor size, parametrial/pelvic wall invasion, hydronephrosis, vascular encasement, bladder/intestine/vagina invasion with their detailed location, lymph node status, stage (I–IV), and enhancement pattern. This study therefore aims to determine the diagnostic efficacy of ADC values for accurate cervical cancer staging and their correlations with this comprehensive tumor characteristic set.

## 2 Methods

This retrospective cross-sectional study was conducted in the Imam Khomeini Hospital Complex center. The study protocol was approved by the institutional review board, and informed consent was waived. Data anonymity was guaranteed through confidentiality agreements signed by all authors, and the study was conducted in accordance with the ethical principles of the Declaration of Helsinki. The study consecutively enrolled a diverse patient population referred to our tertiary center between January 2021 and January 2024. The inclusion criteria were [1]: pathologically confirmed cervical cancer [2], access to the patient’s demographic and past medical history, and [3] availability of pelvic MRI with DWI images performed at our institution. Additionally, the exclusion criteria were as follows [1]: history of any gynecological cancer [2], previous radiotherapy, chemotherapy, or surgery for the treatment of any gynecological cancers [3], history of claustrophobia [4], any contraindications for MRI, and [5] lack of MRI images with sufficient quality. The quality of MRI images was deemed sufficient when MRI scans exhibited an appropriate field of view and adequate anatomical coverage with no significant artifacts. A total number of 146 patients were finally participated in our analysis.

According to our institution’s protocol for cervical cancer staging, enhanced MRI imaging was performed for all patients without any contraindications. All patients were required to fast for at least three hours before imaging. The imaging was performed by a three-Tesla MRI device (GE 3 T discovery 750Gem, phase array coil surface). The protocol of imaging included standard T1-weighted in the axial plane, T2-weighted turbo spin-echo sequences and DWI in the axial and sagittal planes, and dynamic contrast-enhanced (DCE) imaging with T1-weighted fat-suppressed volumetric interpolated breath-hold examination (VIBE) and gadolinium-enhanced T1-weighted sequences. The DWI was obtained in the axial plane with b-values of 0, 500, and 1000 s/mm2. Moreover, to investigate the correlation between ADC values and tumor stage, regions with high enhancement on DWI were identified. ADC maps were automatically generated on the scanner’s console for each patient using a mono-exponential model fitted to the acquired diffusion-weighted images (b-values: 0, 500, and 1000 s/mm²). All maps were subsequently transferred to a dedicated post-processing workstation (AW 4.7, GE Healthcare). To minimize the influence of noise and artifacts, the ADC maps were qualitatively reviewed for alignment with the anatomical T2-weighted sequences and checked for obvious distortion. No additional image filtering was applied. Additionally, before imaging, patients received Hyoscine methyl bromide in order to reduce peristalsis.

Two highly experienced radiologists assessed the MRI images. They were blinded to the patient’s clinical and biochemical circumstances. Tumor staging was determined according to the 2018 FIGO Staging Classification. A region of interest (ROI) measuring at least 1 cm in diameter was manually delineated within each tumor, avoiding areas of necrosis or hemorrhage to minimize bias and ensure accurate representation of solid tumor regions. Discrepancies in the initial independent ROI placement and staging assessments were resolved through a consensus review. Both radiologists jointly re-examined the images to define tumor borders and imaging characteristics, applying recognized staging criteria, until a final, agreed-upon ROI and stage were determined for each case. Mean ADC values within ROI of each patient were automatically computed on the workstation and recorded for subsequent correlation with tumor staging and other clinicopathological features.

Statistical analyses were conducted using R software version 4.3.2. Descriptive statistics, including mean, standard deviation, median, frequency, and range (minimum–maximum), were used to summarize the study data. The Shapiro–Wilk test was applied to assess the normality of quantitative variables. For comparisons between two groups, independent *t*-tests were used when data followed a normal distribution, whereas the Mann–Whitney *U* test was applied for non-normally distributed data. In the case of three or more groups, ANOVA was used for normally distributed data, while the Kruskal–Wallis test was performed for non-normally distributed data. Along with unadjusted p-values we reported the pairwise comparisons adjusted using the Benjamini–Hochberg false discovery rate (FDR) correction, which was selected to control for type I error. Spearman’s rank correlation was employed to evaluate the relationship between ADC values and age, stage, and tumor size. Interpretation zones for the Spearman’s rank correlation are as follows: negligible (0.0–0.09), weak (0.1–0.39), moderate (0.4–0.69), strong (0.7–0.89), and very strong (0.9–1.0). The diagnostic performance of the ADC values was assessed by calculating sensitivity, specificity, accuracy, and the area under the curve (AUC) from the receiver operating characteristic (ROC) analysis. The optimal ADC cutoff value for differentiating tumor stages was determined using the Youden index.

## 3 Results

A total of 146 patients were enrolled in our study with a median age of 48.0 years (IQR 18.75, 28.0–77.0). Between the two age groups of patients (≥40 n: 104, <40 n: 38), the median ADC values were not significantly different (≥40 years = 880.5 ×10⁻⁶ mm²/s, <40 = 799.0 ×10⁻⁶ mm²/s, p = 0.169) and there was no correlation between ADC values and age (Rho = −0.036, p = 0.684). The median tumor size in our study was 48.0 mm (IQR 34.0, 50.0–155.0) and there was a negative correlation between tumor size and ADC values (Rho = −0.25, p = 0.003), as larger tumors were associated with marginally insignificant lower ADC values (≥4 cm = 791.0, <4 cm = 891 ×10⁻⁶ mm²/s, p < 0.001). Two patients presented with distant metastases; one patient had lung metastasis, which was determined by reviewing medical records. One patient had bone metastasis in the pelvis.

**Fig. 1:**
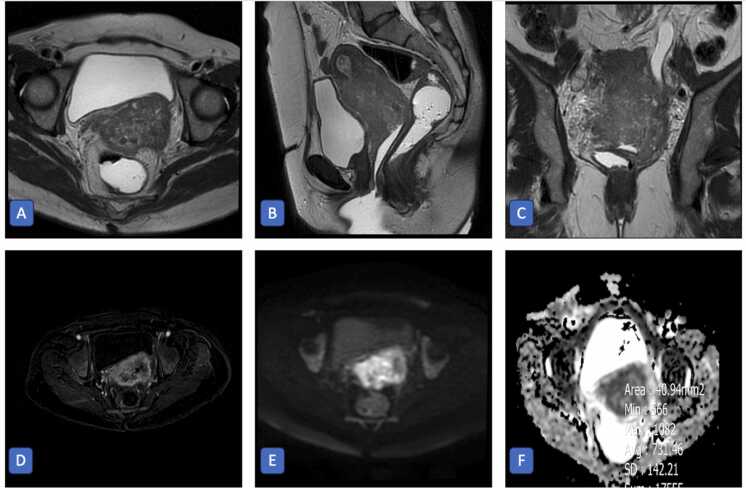
Axial (A), sagittal (B), and coronal (C) T2-weighted, subtraction T1-weighted post-contrast (D), axial diffusion-weighted (E), and ADC (F) MR images of a 57-year-old woman with cervical carcinoma. The tumor measures approximately 76 mm in craniocaudal length and demonstrates parametrial and proximal vaginal invasion, involvement of the left ureterovesical junction with resultant left hydroureteronephrosis, and direct invasion of the adjacent bladder wall. Restricted diffusion with a low ADC value of 731 × 10⁻⁶ mm²/s is observed, consistent with FIGO stage IVA cervical carcinoma.

**Fig. 2:**
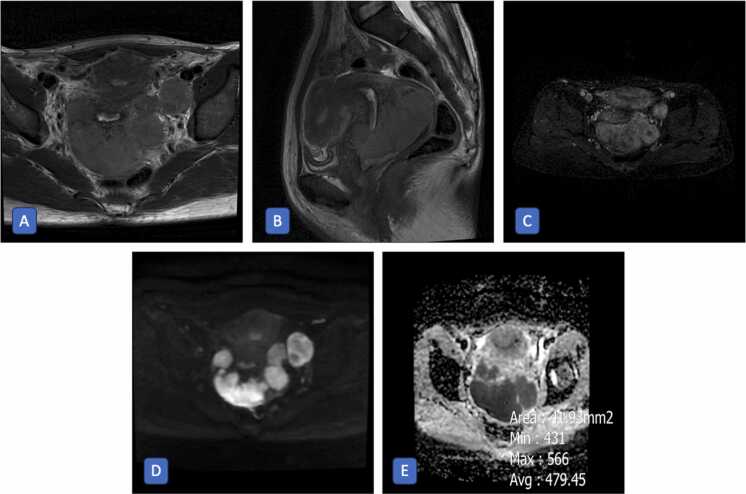
Axial T2-weighted (A), sagittal T2-weighted (B), subtraction T1-weighted post-contrast (C), axial diffusion-weighted (D), and apparent diffusion coefficient (ADC) (E) MR images of a 43-year-old woman with biopsy-proven cervical carcinoma. The images show a large cervical mass measuring approximately 97 mm in maximum diameter with parametrial and proximal vaginal extension A prominent left external iliac lymphadenopathy is also present, showing restricted diffusion on DWI. The primary cervical tumor demonstrates marked diffusion restriction with a corresponding low ADC value of 471 × 10⁻⁶ mm²/s, findings compatible with FIGO stage IIIC1 cervical carcinoma.

Although parametrial invasion (yes n:107, no n:39) was associated with lower ADC values (yes = 787.0, no = 656.0 ×10⁻⁶ mm²/s, p < 0.001), pelvic wall, bladder and intestine invasion and their locations did not correlate with ADC values (p > 0.05) (Table 1). Additionally, no significant difference in median ADC value was found between the absence or presence of hydronephrosis (presence = 789.0, absence = 829.0 ×10⁻⁶ mm²/s, p = 0.569) and its location (left = 705.5, right = 901.0, both: 771.0 ×10⁻⁶ mm²/s, p = 0.102) (Fig. 1). Meanwhile, the existence of vaginal invasion was associated with a lower ADC value (invasion = 809.0, no invasion = 919.0 ×10⁻⁶ mm²/s, p = 0.0199). However, the location of vaginal invasion (proximal, middle, or distal) did not significantly affect ADC values (p = 0.134). It is worth mentioning that with regard to the investigation of lymph node involvement, the median ADC values of metastatic and non-metastatic lymph nodes were 785 × 10⁻⁶ mm²/s and 867 × 10⁻⁶ mm²/s respectively and the difference was significant (p = 0.027) (Fig. 2).

**Table 1:** The median ADC values between factors affecting cervical cancer staging according to FIGO classification.

	No. of patients (%)	Median ADC Value (IQR) (x 10^−6^ mm^2^/s)	Unadjusted p-value	FDR-adjusted p-value
*Tumor size (cm)*				
*< 4*	54 (36.99%)	891.0 (275.5)		
*≥ 4*	90 (61.64%)	791.0 (253.75)	*0.010*	0.057
*Missing*	2 (1.37%)			
*Parametrial invasion*				
*Yes*	107 (73.29%)	787.0 (255.25)	*0.001 >*	0.002
*No*	39 (26.71%)	956.0 (179.0)		
*Pelvic wall invasion*				
*Yes*	13 (8.90%)	728.0 (196.0)		0.278
*No*	133 (91.10%)	829.5 (294.0)	0.204	
*Hydronephrosis*				
*Yes*	20 (13.70%)	789.0 (201.0)		
*No*	126 (86.30%)	829.0 (302.5)	0.569	0.569
*Hydronephrosis location*				
*Left*	9 (45.0%)	705.5 (219.25)		
*Right*	4 (20.0%)	901.0 (288.0)	0.102	0.191
*Both*	7 (35.0%)	771.0 (157.0)		
*Vascular encasement*				
*Yes*	1 (0.69%)	675.0 (0)		0.472
*No*	145 (99.31%)	829.0 (283.25)	0.435	
*Bladder invasion*				
*Yes*	39 (26.71%)	815.0 (210.0)		
*No*	107 (73.29%)	829.0 (320.0)	0.440	0.472
*Bladder invasion location*			ANOVA	
*Serosa*	9 (23.08%)	892.30 (123.0)	0.066	0.153
*Serosa, Muscular*	8 (20.51%)	703.5 (194.25)		
*Serosa, Muscular, Mucosa*	22 (56.41%)	771.0 (219.0)		
*Intestine invasion*				
*Yes*	33 (22.60%)	812.0 (206.5)		0.240
*No*	113 (77.40%)	829.0 (301.25)	0.176	
*Intestine invasion location*			ANOVA	
*Serosa*	15 (45.46%)	771.0 (186.0)	0.071	0.153
*Serosa, Muscular*	10 (30.30%)	843.0 (93.0)		
*Serosa, Muscular, Mucosa*	8 (24.24%)	653.0 (244.0)		
*Vagina invasion*				
*Yes*	109 (74.66%)	809.0 (266.0)		
*No*	37 (25.34%)	919.0 (327.0)	*0.019*	0.074
*Vagina invasion location*				
*Proximal*	88 (80.73%)	828.0 (246.0)		
*Middle*	15 (13.76%)	703.0 (243.5)	0.134	0.224
*Distal*	6 (5.51%)	668.0 (288.75)		
*Lymph node*				
*Yes*	66 (45.21%)	785.0 (229.0)		
*No*	80 (54.79%)	867.0 (299.0)	*0.027*	0.081
*Stage*				
*I*	19 (13.01%)	966.0 (181.5)		
*II*	47 (32.19%)	830.0 (286.75)	*0.001 >*	0.004
*III*	57 (39.05%)	810.5 (229.5)		
*IV*	23 (15.75%)	749.5 (230.75)		
*Enhancement*				
*Heterogeneous*	8 (5.48%)	924.5 (336.5)		
*Homogeneous*	135 (92.47%)	827.0 (280.0)	0.158	0.230
*Missing*	3 (2.05%)

Tumor staging is one of the key stones for choosing proper treatment of cervical cancer. In stage IA, IB, and IIA, surgery can be the treatment of choice for patients and in higher grades it might not be performed. In our study, cervical lesions through stage I to IV were detected (I n: 19, II n: 47, III n: 57, IV n: 23) and tumor stage was negatively correlated with ADC values (Rho = −0.266, p = 0.002). As a result, advanced stages were associated with significantly lower median ADC values (I = 996.0, II = 830.0, III = 810.5, IV = 749.5 ×10⁻⁶ mm²/s, p < 0.01).

Based on ROC curve analysis, with an ADC value cut-off of 923.5 × 10⁻⁶ mm²/s, lesions ≥ 4 cm can be distinguished from those < 4 cm with an accuracy of 0.70 (95% CI: 0.62–0.78) and sensitivity and specificity of 0.82 (95% CI: 0.55–0.93) and 0.47 (95% CI: 0.33–0.76), respectively (AUC = 0.63, 95% CI: 0.53–0.74). Additionally, stage I tumors were accurately distinguished from advanced stages using ADC value (AUC = 0.82, 95% CI: 0.69–0.95) with a sensitivity, specificity and accuracy of 0.80 (95% CI: 0.72–0.90),0.84 (95% CI: 0.67–0.1.0), and 0.81 (95% CI: 0.74–0.89) respectively. Moreover, advanced stages (≥ IIB) were diagnosed from early stages (≤ IIA) with a sensitivity, specificity, and accuracy of 0.82 (95% CI: 0.69–0.90),0.65 (95% CI: 0.46–0.85), and 0.79 (95% CI: 0.69–00.86), respectively (AUC = 0.69, 95% CI: 0.56–0.83). An overview of ADC value diagnostic performance in parametrial, lymph node, and vaginal invasion is shown in detail in Table 2.

**Table 2:** Sensitivity and specificity for the detection of parametrial infiltration, involvement of lymph nodes and vaginal infiltration using ADC values.

	Threshold	Sensitivity (95% CI)	Specificity (95% CI)	Accuracy (95% CI)	AUC (95% CI)
Parametrial involvement	938.5	0.850 (0.570 – 0.920)	0.580 (0.193 – 0.741)	0.771 (0.603–0.848)	0.726 (0.616–0.836)
Lymph node involvement	841.5	0.688 (0.360–0.819)	0.571 (0.371–0.700)	0.633 (0.564–0.717)	0.612 (0.515–0.709)
Vaginal involvement	893	0.722 (0.356 – 0.851)	0.566 (0.300–0.733)	0.717 (0.534–0.832)	0.640 (0.518–0.762)

## 4 Discussion

Despite major improvements in the screening and prevention of cervical cancer, it still remains a great cause of morbidity and mortality worldwide. Proper staging can advance patient prognosis by choosing suitable treatment. By diagnosing cervical cancer at an early stage, it can be treated by local surgical removal compared to more advanced tumors, which require chemoradiotherapy or adjuvant therapy [13], [14]. Moreover, MRI can detect major prognostic features of cervical lesions such as parametrial invasion, pelvic wall infiltration, and lymph node metastasis with higher sensitivity and specificity superior to a CT scan [15], [16]. In this study, we evaluate the role of ADC values in the preoperative staging of cervical cancer.

Based on our analysis, the median ADC values in tumors with a size ≥ 4 cm were significantly lower than those with a size < 4 cm (p = 0.003). This result was consistent with previous literature. For instance, Liu et al. similarly observed that the mean ADC value in tumors ≥ 4 cm was significantly lower than that of smaller lesions [7]. The precise evaluation of tumor size and extent afforded by DWI and ADC mapping is critical, as it directly reduce the risk of over-staging. Over-staging, carries substantial adverse consequences for patient management, leading to overtreatment, wherein patients are subjected to unnecessarily aggressive therapies such as chemoradiation instead of curative surgery. This only exposes patients to the associated morbidities—including chronic bowel and bladder dysfunction, vaginal stenosis, and premature menopause—without any additional survival benefit [17], [18]. Furthermore, the precise anatomic and functional data provided by DWI and ADC mapping are valuable for identifying young patients with cervical cancer who are optimal candidates for fertility-sparing treatment strategies. For these patients, a primary surgical approach like radical trachelectomy can offer oncologic safety with the possibility of future pregnancy. By accurately excluding patients with more extensive disease (e.g., tumors ≥4 cm or with parametrial invasion) who would require standard radical hysterectomy or chemoradiation, DWI ensures that only those with a true low-risk profile are directed towards fertility-preserving protocols. In more advanced cases still confined to the cervix, the identification of larger tumors via DWI can also facilitate a neoadjuvant chemotherapy approach, with the goal of tumor reduction to downstage the disease and potentially make a patient eligible for subsequent fertility-sparing surgery. Thus, the imaging parameters derived from our study are not merely diagnostic but are fundamentally integrated into a personalized treatment algorithm, where preserving reproductive potential is a key quality-of-life outcome [17], [18]. Furthermore, the prognostic significance of DWI was reinforced by Exner et al., who demonstrated its value in predicting long-term treatment success and recurrence risk [5].

Consistent with the underlying pathophysiology, we observed a progressive reduction in ADC values with advancing disease stage. McVeigh et al. reported that with regard to FIGO classification, the mean ADC values in stage IB/IIA are lower than in stage IIB/III/IV [19]. The lower ADC values observed in more advanced tumors might be attributed to higher cellularity and cellular density [14], [20], [21]. Payne et al. reported that high-grade tumors, characterized by increased cellular density, exhibited restricted water diffusion, resulting in lower ADC values. They also demonstrated that poorly differentiated tumors show significantly lower ADC values compared to well-differentiated ones [22].

In our study, ADC mapping demonstrated a moderate discriminative value with a sensitivity of 78.4% and a specificity of 86.6% in detecting stage I from other stages. Additionally, advanced stages (≥ IIB) were differentiated by ADC values with a sensitivity of 80% and a specificity of 65.3% further reflecting its moderate diagnostic performance. In line with our findings, Bhardwaj et al. reported that ADC mapping differentiated Stage I with a sensitivity of 90.9% and a specificity of 70.6%, stage II with a sensitivity of 68.2% and a specificity of 30.4%, stage III with a sensitivity of 66.7% and a specificity of 38.1%, and stage IV with a sensitivity of 66.7% and a specificity of 22.2% [23]. Collectively, our results alongside the existing literature confirm that while ADC mapping provides valuable quantitative data for staging, its moderate performance (mainly due to lower specificities) indicates it should be integrated with other conventional MRI sequences rather than used as a standalone diagnostic tool.

Detecting lymph node involvement in cervical cancer is critically important, as it has a direct influence on treatment strategy and tumor staging. DWI might improve the diagnostic accuracy of metastatic lymph nodes as they tend to have lower mean ADC values compared to benign ones [24]. Our results showed that ADC values can detect lymphatic involvement with an accuracy of 61%, a sensitivity of 68.8%, and a specificity of 57.1%. Although, in contrast to our findings, Rizzo et al. found no significant association between DWI parameters and lymph node metastasis, Chen et al. reported that DWI with ADC measurements could differentiate metastatic lymph nodes from hyperplastic ones with an accuracy, sensitivity, and specificity of 78.4%, 83.3%, and 74.4%, respectively [25], [26]. In another study, Liu et al. reported a sensitivity of 95.7% and a specificity of 96.5% in differentiating metastatic lymph nodes from non-metastatic ones [7]. A systematic review and meta-analysis by Shen et al. concluded that DWI offers high accuracy with a pooled sensitivity of 86% and a specificity of 84%. Additionally, different results were related to variability in ADC cut-off values, which was deemed to be associated with differences in instrument manufacturers, magnetic field, and sequences [27].

Parametrial invasion is a defining criterion for stage IIB of cervical cancer and can be detected by conventional MRI with a pooled sensitivity and specificity of 71–75% and 91–92%, respectively [28], [29]. A systematic review by Dappa et al. reported that conventional T2-weighted MRI imaging could overestimate parametrial extension, particularly in large tumors, due to compression effects or inflammation in stromal tissues [30]. In our study, parametrial invasion was detected using DWI and ADC values with a sensitivity of 85%, a specificity of 58%, and an accuracy of 78.6%. On the contrary, Park et al. concluded that the fusion of DWI and T2 MRI could significantly increase diagnostic accuracy [31]. It was also noted that this combination of T2 MRI and DWI might increase the diagnostic accuracy of parametrial invasion over peritumoral edema [24].

It is worth mentioning that the DWI sequence proved highly informative and reliable in preoperative cervical cancer staging, with qualitative evaluations showing no significant differences between full-protocol MRI (including all sequences and contrast-enhanced images) and a modified abbreviated protocol (T2-weighted sequences plus DWI/ADC maps) based on the 2018 FIGO classification. In a retrospective analysis of 128 patients, both protocols assigned identical numbers to stage I (A/B) and stage IIB, with minor discrepancies in stage II (two more IIA cases in full protocol) and stage III (three more IIIA/B and five fewer IIIC in abbreviated protocol), alongside four additional IVA cases in the abbreviated protocol. Also, overall weighted agreement was excellent (kappa 0.967). Organ involvement assessments demonstrated very good concordance (kappa 0.895–0.983), detecting comparable rates of bladder (22.9–25%), bowel (21.1–21.9%), uterine (48.8–52.8%), and lymph node (47.8–52.2%) involvement without significant differences (McNemar p > 0.05 across all). Although abbreviated protocols occasionally resulted in upstaging (particularly in tumor staging and assessment of certain invasions), these discrepancies were not statistically significant, underscoring DWI's pivotal role in maintaining staging accuracy without contrast and supporting its utility for efficient, cost-effective MRI evaluation in selected populations [3].

The primary strength of ADC mapping lies in its ability to provide objective, reproducible metrics that correlate with disease stage and certain high-risk features like lymph node metastasis and parametrial involvement, offering a potential tool to augment traditional morphological assessment. However, key limitations temper its standalone utility. It is notable that in our cohort, ADC values did not show a statistically significant difference in patients with or without specific local invasions, including hydronephrosis and involvement of the pelvic sidewall, bladder, intestine, or vasculature. Data in the literature regarding ADC’s role in evaluating these features remain scarce. However, a retrospective series of 40 patients found no significant difference in tumor ADC values between cervical cancer patients with and without locoregional (adjacent organ) invasion in parallel to our results, suggesting that ADC may have limited value for assessing specific local extension such as bladder or rectal involvement [32]. Our results suggest that while ADC provides valuable quantitative information for certain staging parameters, its utility for assessing some local invasive status is limited. Therefore, ADC is best positioned not as a replacement for conventional MRI sequences or FIGO staging, but as a complementary biomarker within a multi-parametric imaging framework.

Our study has its own limitations. (1) The absence of histopathologic confirmation of staging in non-surgical cases introduces a form of verification bias, as the reference standard was not uniformly applied across all patients. In our cohort, postoperative histopathology as the gold standard for assessing local extension was available mainly for early-stage patients who underwent surgery, representing only a small subset of the study population. In more advanced cases managed non-surgically, staging and organ involvement were verified solely by imaging and clinical assessment, which may differ from true histopathologic findings. This partial and non-uniform verification could have influenced the estimated diagnostic performance of DWI, potentially leading to either over- or underestimation of its accuracy. (2) Conducting this investigation as a retrospective, single-center study introduces potential selection bias and reflects the imaging practices and treatment pathways of only one institution, which may limit the generalizability of our findings. Future multicenter, prospective studies with larger and more diverse patient populations are needed to confirm these observations and enhance the robustness and external validity of our results. (3) The determination of the final staging and delineation of ROIs were both based on consensus between the two radiologists. Consequently, we could not quantify the inter-observer agreement for these assessments using statistical measures such as Cohen's kappa or the intraclass correlation coefficient. Future studies employing independent assessments would allow for this valuable reliability analysis.

In conclusion, our study showed that lower ADC values in DWI sequences in the preoperative staging are associated with a higher tumor stage. This study demonstrated that DWI and median ADC values could differentiate high-grade tumors from low-grade ones and indicate the aggressiveness of a tumor by demonstrating lymph node metastasis and parametrial involvement with adequate sensitivity and specificity.

## Clinical trial number

N/A

## Authors contribution

All authors contributed significantly to this article. PKH: data interpretation, data collection. FZ: manager and principal investigator, data collection. SA: primary writer of manuscript. SM: review and editing, FMS: statistical analysis. HZ: figures, writing. MH: data interpretation

## CRediT authorship contribution statement

**Fahimeh Zeinalkhani:** Supervision, Project administration, Data curation, Conceptualization. **Saeed Mohammadzadeh:** Writing – review & editing, Writing – original draft, Supervision, Data curation. **Soroush Alaeddini:** Validation, Supervision, Data curation. **Hadise Zeinalkhani:** Supervision, Data curation, Conceptualization. **Fatemeh Mahdavi Sabet:** Writing – review & editing, Writing – original draft, Supervision, Data curation. **Mahdi Hazratgholi:** Supervision. **Peyman Kamali Hakim:** Writing – review & editing, Writing – original draft, Formal analysis, Data curation, Conceptualization.

## Human ethics and consent to participate

All participants provided written informed consent. We adhered to the principles outlined in the Declaration of Helsinki. The institutional review board of the BLINDED approved our study.

## Ethical statement

This study was approved by the institutional review board of the authors' institution. All participants provided written informed consent, and the study was conducted in accordance with the ethical principles of the Declaration of Helsinki.

## Animal study

N/A

## Funding

The authors received no financial support for the research, authorship, and/or publication of this article.

## Declaration of Competing Interest

We, the undersigned authors, hereby declare that we have no financial or personal relationships with other people or organizations that could inappropriately influence or bias the content of this work.

Specifically:

• We have no financial interests (including employment, consultancies, stock ownership, honoraria, patents, etc.) related to this research
• We have no professional affiliations that could be perceived as influencing the results presented in this manuscript
• No funding sources were involved in the preparation of this study
• All authors have reviewed this declaration and confirm its accuracy

The authors alone are responsible for the content and writing of this paper.

## Data Availability

The data supporting this article can be obtained from the corresponding author upon request.
